# 苯达莫司汀联合利妥昔单抗一线治疗非滤泡性淋巴瘤的B淋巴细胞增殖性疾病临床分析

**DOI:** 10.3760/cma.j.cn121090-20260226-00109

**Published:** 2026-07

**Authors:** 欣然 秘, 文婕 熊, 禹廷 阎, 婷玉 王, 刚 安, 德慧 邹, 燕 徐, 薇 刘, 录贵 邱, 瑞 吕, 树华 易

**Affiliations:** 1 天津医科大学，细胞生态海河实验室，天津 300070 Haihe Laboratory of Cell Ecosystem, Tianjin Medical University, Tianjin 300070, China; 2 中国医学科学院血液病医院（中国医学科学院血液学研究所），血液与健康全国重点实验室，国家血液系统疾病临床医学研究中心，细胞生态海河实验室，天津 300020 State Key Laboratory of Experimental Hematology, National Clinical Research Center for Blood Diseases, Haihe Laboratory of Cell Ecosystem, Institute of Hematology & Blood Disarses Hospital, Chinese Academy of Medical Sciences & Peking Union Medical College, Tianjin 300020, China; 3 天津医学健康研究院，天津 301600 Tianjin Institutes of Health Science, Tianjin 301600, China

## Abstract

为明确苯达莫司汀联合利妥昔单抗一线治疗非滤泡性淋巴瘤（FL）的B淋巴细胞增殖性疾病（BLPD）疗效及安全性，依托国家血液系统疾病队列研究-淋巴瘤队列，纳入于2019年8月至2025年2月在中国医学科学院血液病医院应用苯达莫司汀联合利妥昔单抗作为一线治疗的初诊非FL的BLPD患者91例。中位年龄59（30～79）岁，男女比例为1.6∶1（56/35）。其中共78例患者接受了≥2个周期的诱导治疗，完全缓解（CR）率为59.0％，客观缓解率为88.5％。主要血液学不良反应包括3～4级中性粒细胞减少、淋巴细胞减少、贫血、血小板减少；非血液系统不良反应包括发热及感染、肝功能异常等。中位随访41（2～70）个月，5年无进展生存（PFS）率为78.1％（95％ *CI*：50.3％～91.5％），5年总生存率为85.2％（95％ *CI*：56.8％～95.6％）。年龄≥60岁是PFS的独立危险因素（*HR*＝9.161，95％ *CI*：1.063～78.960，*P*＝0.044），疗效评估达到CR是PFS的独立保护因素（*HR*＝0.087，95％ *CI*：0.009～0.807，*P*＝0.032）。苯达莫司汀联合利妥昔单抗可作为非FL的BLPD患者一线治疗的有效选择。

## Abstract (en)

This study aimed to evaluate the efficacy and safety of bendamustine combined with rituximab as first-line therapy in B-cell lymphoproliferative disorders（BLPD）other than follicular lymphoma（FL）. Based on the Lymphoma Subcohort of the National Hematological Disease Cohort Study, a total of 91 newly diagnosed patients with non-FL BLPD receiving frontline bendamustine-rituximab regimen at the Institute of Hematology & Blood Diseases Hospital, Chinese Academy of Medical Sciences from August 2019 to February 2025 were enrolled.The median age of the cohort was 59 years（range, 30–79 years）, with a male-to-female ratio of 1.6:1（56 males and 35 females）. Among all subjects, 78 patients completed ≥2 cycles of induction therapy, with a best complete response（CR）rate of 59.0％ and an objective response rate of 88.5％. The primary grade 3–4 hematological adverse events included neutropenia, lymphopenia, anemia and thrombocytopenia, while common non-hematological adverse events consisted of fever, infection and abnormal liver function. With a median follow-up duration of 41 months（range, 2–70 months）, the 5-year progression-free survival（PFS）rate and 5-year overall survival（OS）rate were 78.1％（95％ *CI*:50.3％–91.5％）and 85.2％（95％ *CI*:56.8％–95.6％）, respectively. Multivariate analysis identified age ≥60 years as an independent risk factor for PFS（*HR*＝9.161，95％ *CI*: 1.063–78.960，*P*＝0.044）, and CR achievement as an independent protective factor for PFS（*HR*＝0.087, 95％ *CI*: 0.009–0.807, *P*＝0.032）. In conclusion, bendamustine combined with rituximab is an effective first-line treatment option for patients with non-FL BLPD.

BR方案（苯达莫司汀+利妥昔单抗）作为经典的免疫化疗方案，在滤泡性淋巴瘤（FL）一线治疗中的循证医学证据已得到充分积累，其疗效与安全性得到广泛认可，奠定了其一线治疗的核心地位。BRIGHT研究的长期随访数据进一步证实，相较于R-CHOP或R-CVP方案，BR方案具有更优的长期疾病控制效果[1]。但对除FL外的B淋巴细胞增殖性疾病（BLPD）患者，尤其是国内患者的疗效及安全性值得更多数据证实。随着无化疗方案在老年惰性淋巴瘤患者中的探索越来越多，长期靶向药物应用后感染的问题逐渐突出，且部分患者无法长期耐受。因此，本研究分析BR方案在除FL以外的BLPD患者（包括老年患者）中的应用，旨在为后续缩短靶向药物应用时长，实现以化疗与靶向药物组合的有限周期治疗的可行性及可能疗程提供思路。

## 病例与方法

1. 病例：本研究为回顾性队列研究，为国家血液系统疾病队列研究-淋巴瘤队列的一部分（NICHE，NCT04645199），纳入2019年8月至2025年2月在中国医学科学院血液病医院（中国医学科学院血液学研究所）应用BR方案作为一线治疗的初诊非FL的BLPD［包括老年套细胞淋巴瘤（MCL）］患者共91例。纳入标准：治疗前进行组织活检，病理诊断符合世界卫生组织（WHO）2022年淋巴组织肿瘤分类标准[2]；既往未接受过治疗。收集患者基线数据包括：性别、初诊年龄、美国东部肿瘤协作组（ECOG）体能状态评分、B症状（乏力、盗汗、体重减轻）、血清LDH、血β_2_微球蛋白、有无淋巴结肿大、有无结外受累、TP53有无异常等。本研究经中国医学科学院血液病医院伦理委员会批准（批件号：IIT2021030-EC-1）并获得患者知情同意。

2. 治疗方案与疗效评估：利妥昔单抗375 mg/m^2^，第0天；苯达莫司汀90 mg/m^2^，第1～2天，28 d为1个周期。定义至少接受2个周期治疗为规范治疗。化疗结束后疗效评估达到部分缓解（PR）及以上的患者，根据患者意愿予利妥昔单抗维持治疗。患者分别于完成2、4、6个周期治疗后应用CT或PET-CT进行疗效评估，并根据2014版Lugano疗效评定标准[3]分为完全缓解（CR）、PR、疾病稳定（SD）和疾病进展（PD）；客观缓解率（ORR）为CR率（CRR）与PR率之和。对于慢性淋巴细胞白血病/小淋巴细胞淋巴瘤（CLL/SLL）患者，参考2018版国际慢性淋巴细胞白血病研讨会指南[4]进行疗效评估。对于淋巴浆细胞淋巴瘤/华氏巨球蛋白血症（LPL/WM）患者，参考第六届华氏巨球蛋白血症国际研讨会更新的标准[5]进行疗效评估。

3. 安全性评价：根据《常见不良反应事件评价标准（CTCAE）》5.0版，对患者接受治疗后发生的不良事件（AE）进行分级。

4. 随访：通过查阅电子病历及拨打电话进行随访。末次随访时间为2025年10月7日。无进展生存（PFS）期定义为患者启动治疗至疾病进展或复发或死亡或末次随访的时间。总生存（OS）期定义为患者启动治疗至因任何原因判定临床死亡或末次随访的时间。

5. 统计学处理：应用SPSS 25.0和Graphpad prism 10软件进行统计学分析。计数资料用例数（百分比）表示，计量资料用*M*（范围）表示。使用卡方检验比较临床特征差异，Kaplan-Meier计算生存率，并用Cox回归分析模型进行生存分析。*P*<0.05为差异具有统计学意义。

## 结果

1. 临床特征：91例患者中男性56例、女性35例，中位年龄59（30～79）岁，≥60岁患者44例（48.4％），ECOG评分0～1分患者占97.8％（89/91），ECOG评分2分患者2例（2.2％）。91例患者中，边缘区淋巴瘤（MZL）51例（56.0％），LPL/WM 12例（13.2％），BLPD，不能分类（BLPD-U）7例（7.7％），CLL/SLL 8例（8.8％），MCL 7例（7.7％），毛细胞白血病变异型（HCL-V）4例（4.4％），冷凝集素病（CAD）2例（2.2％）。伴B症状的患者25例（27.5％），伴有骨髓侵犯的患者68例（74.7％），结外病灶>1处的患者29例（31.9％），血β_2_微球蛋白升高的患者69例（75.8％），血清LDH升高的患者28例（30.8％），TP53异常的患者10例（10.9％）。在MZL及LPL中，分别以MZL-IPI评分以及WM的国际预后评分系统为据进行危险分层，中高危组患者分别占33.3％（17/51）及25.0％（3/12）。

78例（85.7％）患者接受规范治疗，按照是否完成4个周期分为≥4个周期组（69例）和<4个周期组（9例），比较上述临床特征，<4个周期组患者年龄≥60岁比例更高［77.8％（7/9）对39.1％（27/69），*P*＝0.033］，其余差异均无统计学意义（*P*值均>0.05）。

2. 疗效评估：入组的91例患者中，可评估疗效有78例，其中69例完成≥4个周期、52例完成6个周期。患者总体（包括维持治疗）CRR为59.0％、ORR为88.5％。完成2个周期治疗后CRR为21.8％，4个周期治疗后CRR为42.0％，6个周期治疗后CRR为59.6％，ORR则从2个周期治疗后的65.4％逐步提升至6个周期治疗后的94.2％。按照不同疾病类别的最佳疗效来看，MZL患者（47例）的CRR为57.4％、ORR为87.2％；LPL/WM患者（11例）的CRR为36.4％、ORR为81.8％；CLL/SLL患者（6例）的CRR为66.7％、ORR为100％；MCL患者（5例）的CRR为100％、ORR为100％；BLPD-U患者（4例）的CRR为100％、ORR为100％；HCL-V患者（4例）的CRR为50.0％、ORR为75.0％；CAD患者（1例）的CRR为0、ORR为100％。如表1所示，年龄≥60岁患者的ORR［79.4％（27/34）对95.5％（42/44），*P*＝0.028］及LDH升高患者的CRR［41.7％（10/24）对66.7（36/54），*P*＝0.048］偏低，同时治疗≥4个周期组较<4个周期组不论是CRR还是ORR均明显提高［CRR：65.2％（45/69）对11.1％（1/9），*P*＝0.002；ORR：94.2％（65/69）对44.4％（4/9），*P*<0.001］，其余各项临床特征差异无统计学意义（*P*值均>0.05）。

**表1:** 接受≥2个周期BR方案治疗的非FL慢性B淋巴细胞增殖性疾病患者（78例）不同临床特征的疗效比较

特征	CRR［％（例数/总例数）］	*χ*^2^值	*P*值	ORR［％（例数/总例数）］	*χ*^2^值	*P*值
年龄≥60岁		0.001	0.981		4.836	0.028
是	58.8（20/34）			79.4（27/34）		
否	59.1（26/44）			95.4（42/44）		
骨髓侵犯		1.351	0.245		0.062	0.803
是	55.2（32/58）			87.9（51/58）		
否	70.0（14/20）			90.0（18/20）		
结外病灶>1处		2.933	0.140		0.550	0.458
是	100（4/4）			100（4/4）		
否	56.7（42/74）			87.8（65/74）		
β_2_微球蛋白升高		1.697	0.693		0.004	>0.05
是	55.0（33/60）			88.3（53/60）		
否	72.2（13/18）			88.9（16/18）		
LDH升高		4.292	0.048		0.031	>0.05
是	41.7（10/24）			87.5（21/24）		
否	66.7（36/54）			88.9（48/54）		
TP53基因异常		0.216	0.685		3.025	0.139
是	33.3（3/6）			66.7（4/6）		
否	59.7（43/72）			90.3（65/72）		
治疗周期≥4个		9.633	0.002		19.312	<0.001
是	65.2（45/69）			94.2（65/69）		
否	11.1（1/9）			44.4（4/9）		

**注** BR：苯达莫司汀+利妥昔单抗；FL：滤泡性淋巴瘤；CRR：完全缓解率；ORR：客观缓解率；LDH：乳酸脱氢酶

3. 生存及预后：中位随访41（2～70）个月，5年PFS率为78.1％（95％ *CI*：50.3％～91.5％），5年OS率为85.2％（95％ *CI*：56.8％～95.6％）。中位PFS、OS期均未达到。如表2所示，单因素分析显示年龄≥60岁（*HR*＝8.572，95％ *CI*：1.047～70.195，*P*＝0.045）、疗效评估达到CR（*HR*＝0.085，95％ *CI*：0.010～0.692，*P*＝0.021）与患者PFS相关，而LDH升高与患者OS相关（*HR*＝7.756，95％ *CI*：1.331～45.208，*P*＝0.023）。TP53异常的6例患者5年PFS率为83.3％（95％ *CI*：27.4％～97.5％），且6例患者5年内均持续存活，但由于其例数过少，未纳入多因素分析。将有统计学意义的3个因素（年龄≥60岁、LDH升高、疗效评估达到CR）以及治疗周期≥4个（该因素在疗效方面差异有统计学意义）纳入多因素Cox回归模型，结果提示年龄≥60岁是PFS的独立危险因素（*HR*＝9.161，95％ *CI*：1.063～78.960，*P*＝0.044），疗效评估达到CR是PFS的独立保护因素（*HR*＝0.087，95％ *CI*：0.009～0.807，*P*＝0.032）；其他因素在校正其他混杂因素后，均未显示出独立的预后价值（*P*值均>0.05）。

**表2:** 接受BR方案治疗的非滤泡性淋巴瘤的B淋巴细胞增殖性疾病患者关于生存的Cox回归分析

因素	无进展生存				总生存			
	单因素分析		多因素分析		单因素分析		多因素分析	
	*HR*（95％ *CI*）	*P*值	*HR*（95％ *CI*）	*P*值	*HR*（95％ *CI*）	*P*值	*HR*（95％ *CI*）	*P*值
年龄≥60岁	8.572（1.047～70.195）	0.045	9.161（1.063～78.960）	0.044	6.749（0.807～56.477）	0.078	9.159（0.792～165.871）	0.076
LDH升高	3.329（0.740～14.987）	0.117	2.048（0.387～10.836）	0.399	7.756（1.331～45.208）	0.023	2.407（0.314～18.432）	0.398
治疗周期≥4个	0.212（0.041～1.083）	0.062	0.797（0.122～5.191）	0.812	0.699（0.079～6.193）	0.747	0.364（0.022～6.060）	0.481
疗效评估达到CR	0.085（0.010～0.692）	0.021	0.087（0.009～0.807）	0.032	0.147（0.017～1.272）	0.082	0.123（0.011～1.364）	0.088

**注** BR：苯达莫司汀+利妥昔单抗；LDH：乳酸脱氢酶；CR：完全缓解

4. 不良反应：入组的91例患者在整体一线治疗（包括维持治疗）过程中，主要的血液系统不良反应包括：淋巴细胞减少83例（91.2％），其中3～4级73例（80.2％）；中性粒细胞减少66例（72.5％），其中3～4级25例（27.5％）；贫血65例（71.4％），其中3～4级7例（7.7％）；血小板减少59例（64.8％），其中3～4级28例（30.8％）。主要的非血液系统不良反应包括：发热及感染（32例，35.2％），肝功能异常（32例，35.2％），皮疹（9例，9.9％），恶心及呕吐（4例，4.4％），肠梗阻（2例，2.2％）。接受<4个周期、4个周期及>4个周期化疗患者的4级中性粒细胞减少比例分别为18.2％（4/22）、12.5％（1/8）、3.3％（2/61），*P*＝0.069；4级淋巴细胞减少比例则分别为22.7％（5/22）、25.0％（2/8）、32.8％（20/61），*P*＝0.645。

## 讨论

BR方案在以FL为代表的BLPD中的疗效和地位早已得到确立。然而，既往报道多数以FL为主，针对具有高度异质性的其他类型BLPD，仍缺乏来自中国人群的翔实数据。本研究分析91例接受一线BR方案治疗的非FL的BLPD患者的临床资料，纳入患者以MZL为主，中位年龄59岁，且近50％患者年龄≥60岁，与stiL[6]及BRIGHT[7]研究的年龄构成类似，亦符合BLPD以老年患者为好发人群的临床特征。疗效方面，整体最佳ORR为87.2％，CRR为57.4％。这一数据与StiL NHL1研究中MZL亚组的疗效（ORR 93％）相近[6]，为BR方案作为晚期MZL患者一线治疗提供证据。而在其他亚型中，BR方案亦展现出明显有效性：MCL和CLL/SLL患者ORR均达到100％，其中老年MCL患者的CRR为100％，这与BR方案在多项研究中显示的优于R-CHOP方案的PFS优势相符，同时也是对美国国立综合癌症网络及中国临床肿瘤学会指南推荐的进一步验证[8]–[9]。而LPL/WM患者的CRR相对较低（36.4％），考虑可能与该疾病独特临床特征以及评估标准有关，即WM往往更关注临床症状改善，其疗效评估更多应用微小缓解率（MRR）及ORR指标，而相对较难达到CR[10]–[11]。

随着周期的推进，本研究中患者疗效显著加深：治疗2个周期后，CRR仅为21.8％；完成4个周期诱导治疗时，CRR提升至42.0％；完成6个周期诱导治疗后，CRR达到59.6％。这一动态变化与苯达莫司汀作为双功能烷化剂和嘌呤类似物的独特药理学机制相符，其诱导的DNA损伤和细胞凋亡需要一定的暴露时间和累积剂量以实现肿瘤细胞的深度清除[12]。多因素Cox回归分析表明，年龄≥60岁是PFS的独立危险因素，而获得CR则是PFS的独立保护因素。尽管以上2个因素对OS的预测未达统计学意义，但其*P*值均处于临界水平（0.05～0.1），提示存在潜在的预后趋势，不排除因随访时间或样本量受限而未能显现其远期效应。进一步结合基线特征与疗效分析可见，老年患者中接受<4个周期治疗比例高，其CR率明显偏低；同时，前期疗效分析亦证实，疗程不足与CRR及ORR的降低相关。据此推测，年龄对PFS的负面影响，在一定程度上可能与化疗强度不足导致早期疗效欠佳相关。因此，对于接受BR方案治疗的BLPD患者，特别是老年患者，临床工作的重心之一应是采取积极的支持治疗策略（如规范使用粒细胞集落刺激因子、加强营养支持、预防性抗感染等），以保障其能够耐受并完成至少4个周期的足周期治疗，促使患者生存获益。同时，本研究结果显示血清LDH升高也是OS相关风险因素，反映LDH升高是肿瘤高负荷和高增殖活性的间接标志，但由于受到其他因素影响，在多因素分析中未显示独立统计学意义，尚待进一步研究验证。此外，本研究中TP53基因异常并未得出阳性结果。这与在CLL及MCL中TP53缺失或突变是独立预后不良因素的结论不一致[13]–[14]。分析可能原因：本研究样本量限制，仅10例患者检出异常，其中有生存预后信息6例，统计效力不足；其次，检测方法局限，FISH仅能检测到缺失或扩增，无法发现点突变，而后者同样具有重要功能影响；此外，TP53异常比例在不同BLPD亚型中的预后价值可能不同，且TP53缺失所界定的比例并无规范标准[15]。因此，本研究的阴性结果并不应被解读为TP53异常在非FL的BLPD中无意义，未来亟需开展前瞻性、大样本研究，并采用包括二代测序在内的更加全面的分子检测方法，以明确TP53及其缺失比例在中国BLPD患者中的精准预后与预测价值。

本研究发生的不良反应类型与BR方案已知特征高度吻合[16]–[18]。非血液学不良反应发生率低，耐受性良好。血液学不良反应中淋巴细胞减少发生率最高（91.2％，3～4级80.2％）、中性粒细胞减少次之（72.5％，3～4级27.5％）。中性粒细胞减少和由此引发的感染风险是临床管理的主要挑战。本研究结果显示周期数与3～4级淋巴细胞减少的发生率呈正相关，提示BR方案诱导的是一种深度且长期的B细胞及T细胞免疫抑制状态。这不仅会增加治疗期间的机会性感染风险（如肺孢子菌肺炎、病毒再激活等），还可能影响治疗后的长期免疫重建和疫苗应答。因此，临床实践必须建立系统性的管理策略：治疗前筛查并控制潜在感染；治疗中常规预防性使用粒细胞集落刺激因子、磺胺类药物及抗病毒药物；治疗后定期监测免疫球蛋白水平及淋巴细胞亚群，并对患者进行充分的感染防护教育。此外，本研究发现接受5～6个周期BR方案治疗患者与接受4个周期的患者ORR数值相近，而发生4级淋巴细胞减少的比例较高（32.8％对25.0％）。以上结果提示，在疗效相当的前提下，4个周期BR方案有可能通过减少累积血液学不良反应的发生，成为更优的周期选择。

本研究作为单中心回顾性研究，存在选择偏倚和信息偏倚；疾病亚型分散，部分罕见亚型样本量小，限制亚组结论的普适性。未来研究可重点探索BR方案与新型药物（如布鲁顿酪氨酸激酶抑制剂、BCL2抑制剂、来那度胺等）的联合或序贯应用，同时基于BR方案不同周期的疗效和不良反应，为有限周期的相对短程治疗进一步寻找最佳周期组合的依据。

综上，本研究表明一线BR方案疗效显著，且疗效与周期数正相关，完成足周期（≥4周期）治疗是非FL的BLPD患者获得深度缓解和长期PFS获益的关键。年龄≥60岁和LDH升高是预后不良的因素。治疗相关血液学不良反应，尤其是深度且持久的淋巴细胞减少，是临床安全管理的核心。未来，基于BR方案的优化与联合策略，将是进一步提升BLPD患者疗效的重要研究方向。
